# Research and Preparation of Food Packaging Materials Based on Polypropylene Using Agricultural and Aquatic Waste

**DOI:** 10.3390/ma19214627

**Published:** 2026-10-30

**Authors:** Aigul Bukanova, Fazilat Kairliyeva, Alimzhan Nauashev, Saule Bukanova, Zhannat Kareshova, Igor Makarov, Ivan Levin, Markel Vinogradov, Junlong Song

**Affiliations:** 1 Institute of Petrochemical Engineering and Ecology Named After N. K., Atyrau Oil and Gas University Named After S. Utebayev, M. Baimukhanov Street, 45A, Atyrau 060027, Kazakhstan; bukanova66@mail.ru (A.B.); alimnau@gmail.com (A.N.); sauleshik81@mail.ru (S.B.); zh.kareshova@mail.ru (Z.K.); 2 A. V. Topchiev Institute of Petrochemical Synthesis, Russian Academy of Sciences, 29 Leninsky Prospect, Moscow 119991, Russia; 3 Jiangsu Co-Innovation Center for Efficient Processing and Utilization of Forest Resources, Nanjing Forestry University, Nanjing 210037, China; junlong.song@njfu.edu.cn

**Keywords:** polypropylene, food packaging, agricultural waste, aquatic waste, biogenic calcium carbonate, nanocellulose, chitosan, barrier properties, compatibilization, migration safety

## Abstract

Growing concern over the environmental burden of petrochemical food packaging has directed attention toward agricultural and aquatic residues as secondary raw materials. This review examines polypropylene (PP) composites containing lignocellulosic fillers and biogenic calcium carbonate derived from eggshells and mollusc shells, together with chitosan- and nanocellulose-based coatings. Bulk modification, waterborne coatings and primers, barrier performance, active packaging, and migration safety are analysed in turn. As an organising framework, we propose a functional-separation strategy in which the bulk matrix carries mechanical load, a continuous coating governs mass transfer, and an active layer preserves the packaged food. Fillers are compared in terms of modulus gain and the retention of strength and impact resistance, with explicit acknowledgement of the limits inherent in comparing heterogeneous datasets. Replacing part of the PP with waste-derived material does not, in itself, confer biodegradability, a lower carbon footprint, or suitability for food contact. The principal knowledge gap is the scarcity of studies in which mechanical, barrier, and migration properties are measured on one and the same packaging system. Moderate filler loadings combined with a thin functional coating are identified as a direction warranting further verification, including assessment of recyclability and life-cycle performance.

## 1. Introduction

Low density, chemical and moisture resistance, moderate cost, and well-established processing technologies have made PP one of the dominant polymers in food packaging [1]. Its weaknesses are equally well known: a comparatively poor oxygen barrier, low surface energy, limited compatibility with hydrophilic fillers, and the absence of rapid biodegradation under natural conditions. For this reason, the future of PP packaging depends on lowering virgin feedstock consumption, designing for recyclability, and strengthening collection and recycling infrastructure [2,3]. It should also be stressed that a bio-based origin does not by itself ensure that an alternative polymer will biodegrade in service or impose a smaller environmental burden [4]. Accordingly, the present review is concerned with improving PP rather than presupposing its inevitable replacement.

Research in this area is shaped by two intrinsic constraints of PP. The first concerns barrier performance: oxygen permeability is relatively high, and in mono-material structures even the water-vapour barrier may fall short of what specific foods require. The second is thermodynamic in nature and stems from the incompatibility between the non-polar matrix and hydrophilic fillers of biological origin. Efforts to overcome these constraints have followed two main routes. One route employs residues from agriculture and aquaculture—lignocellulosic biomass, rice husk and its ash, eggshells and mollusc shells, chitosan from crustacean exoskeletons, and nanocellulose from fibre-rich streams—as fillers and functional additives. The other relies on waterborne coatings and primers that upgrade barrier properties and serve as vehicles for active, chiefly antioxidant and antimicrobial, functions.

Agri-food residues constitute a large reservoir of renewable biomass [5] from which fillers and functional components can be sourced for packaging, including PP-based systems [6]. Their properties, however, depend on feedstock origin and on the isolation and pretreatment routes used, which complicates the comparison of results obtained in different laboratories [7].

**Figure 1:**
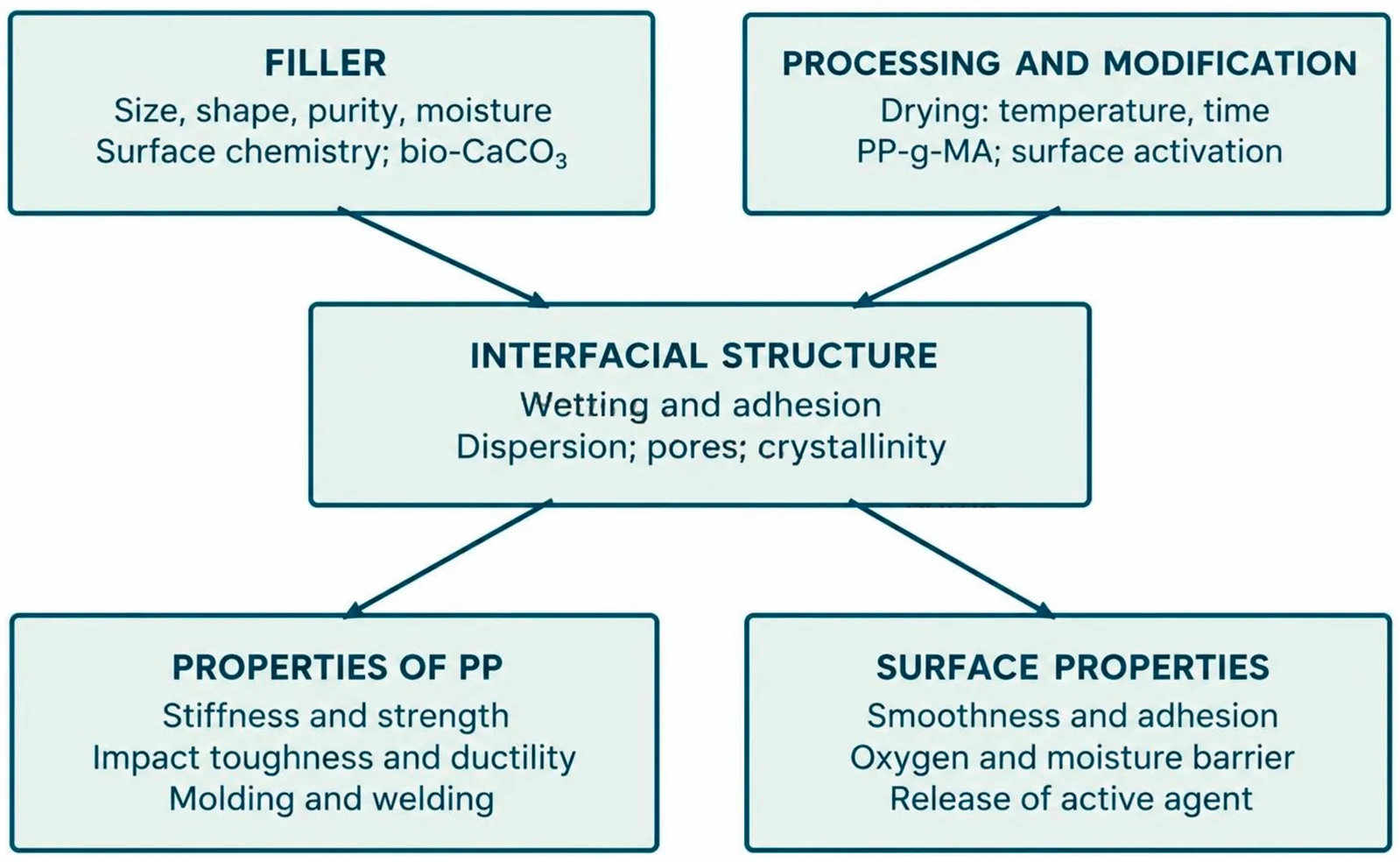
Interrelationships among filler characteristics, surface modification, and processing, and their influence on interfacial structure and the end-use performance of PP packaging. Arrows denote relationships that require experimental verification.

Three gaps define the scope of this review: (i) the shortage of comparable filler data generated within a single PP matrix; (ii) the weak link between laboratory mechanical or barrier metrics and the actual preservation of a given food; and (iii) the lack of combined assessment of migration, recyclability, and life-cycle impact. The distinctive contribution of this work lies in integrating these issues within a functional-separation strategy (Figure 1).

Under the functional-separation strategy, the load-bearing PP bulk, the barrier coating, and the active layer are optimised independently. Lignocellulose and biogenic CaCO_3_ are candidates for tuning stiffness, whereas chitosan and nanocellulose are better suited to surface functions. What this describes is functional complementarity, not a demonstrated synergy arising from combined filling; any claimed synergy should be tested against the individual components at an identical total loading. The modulus-gain normalisation applied in Section 8.1. Concept of Functional Separation is used purely as a descriptive metric and does not eliminate differences between study conditions.

### Literature Selection and Comparison Approach

This work is a critical narrative review. Its core subject is fillers and coatings for PP-based packaging; data obtained for other matrices are drawn upon only where they help explain mechanisms. In comparing studies, we single out formulation, the PP reference, feedstock preparation, processing, and test conditions. Values reported by different studies are not pooled into an overall ranking unless the methods have been reconciled, and information that could not be verified is marked NV. Because a fully reproducible search protocol—specifying databases, dates, search strings, time coverage, and the number of publications retained—was not documented, we make no claim of PRISMA compliance or exhaustive coverage; this is acknowledged as a limitation of the review.

## 2. Bulk Modification: PP Composites Filled with Agricultural and Aquaculture Residues

### 2.1. Lignocellulosic Fillers

A strong incentive for studying this filler class is the sheer volume of plant residues requiring management. According to estimates by Cherubin et al. [8], the proportion of plant residues—straw, litter, sawdust, logging waste, weeds, and other by-products—relative to total production grew by roughly 33% between 2003 and 2013. By 2013, their combined mass exceeded 5 billion t, distributed as 47% in Asia, 29% in the Americas, 16% in Europe, 6% in Africa, and 2% in Oceania. Although landfilling is still the prevailing disposal route, it is becoming increasingly untenable because of deteriorating air quality, the withdrawal of land from productive use, methane emissions, and leachate formation [9,10,11]. These pressures favour deeper valorisation, in which residues are treated as feedstock rather than waste [11].

Both early and recent studies confirm that wood flour, rice husk and its ash, hemp shives, and comparable lignocellulosic materials raise the elastic modulus, thermal stability, and heat resistance of PP. This stiffening, however, frequently comes at the expense of tensile strength and impact resistance whenever interfacial compatibility is inadequate and dispersion is poor. The most reproducible outcome is obtained by pairing maleic anhydride-grafted polypropylene (PP-g-MA, hereafter MAPP) with alkaline or silane treatment of the fibre, a combination that reduces both interfacial defects and moisture uptake [6].

#### 2.1.1. Rice Husk and Rice Husk Ash: Feedstock Availability and Composition

Rice husk (RH), a readily available by-product of rice milling, has been explored as a component of polymer composites since the 1970s [12,13,14,15]. For packaging applications, the critical preparatory factors are drying, particle-size distribution, removal of extractives, and control over silica-bearing particles, which can accelerate equipment wear and introduce defects into thin films.

The husk represents about 20–25% of the rice-grain mass and is notably rich in silica (Table 1). Because the reported compositional ranges reflect a heterogeneous feedstock, the moisture content and the basis on which results are expressed should be specified whenever formulations are calculated.

Rice husk ash (RHA), whose SiO_2_ content can reach 90% [16], is regarded as a potential siliceous filler for PP. Its properties are governed by the combustion regime, residual carbon, purity, and degree of agglomeration. Findings obtained for construction materials cannot be taken as evidence that RHA is suitable for food packaging.

#### 2.1.2. Behavior of RH and RHA in Polymer Matrices

Natural-fibre/PP composites are processed by extrusion, injection moulding, and compression moulding. Because interfacial modifiers affect adhesion and melt rheology simultaneously, RH/PP formulations must strike a balance between achieving good dispersion and avoiding thermal damage to the biophase [17]. Optima established for polystyrene and other matrices do not carry over to PP.

For RH/PP systems, strength improvements were achieved using MAPP as well as PP grafted with m-isopropenyl-α,α-dimethylbenzyl isocyanate [18]. Nevertheless, data from highly filled mechanical specimens say nothing about film drawability, heat sealability, or food-contact safety; each of these attributes requires dedicated testing.

#### 2.1.3. Compatibilization of PP/RH Systems

When non-polar PP meets the polar surface of RH, the resulting incompatibility promotes agglomeration and loss of strength. MAPP enhances interfacial interaction [18]; yet the accompanying change in melt flow is also influenced by its molecular weight, grafting degree, dispersion state, and degradation [19]. Melt-flow index values should therefore be reported together with the test temperature and load, and a decrease in this index alone cannot be taken as proof of chemical bonding.

Work on Vietnamese rice husk fibre (RHF) demonstrated that MAPP concurrently improved tensile and flexural strength, impact resistance, and thermal stability of the biocomposites, confirming its effectiveness at the RHF–matrix boundary; the authors themselves regard Vietnamese RHF as a promising renewable resource [17]. Comparable outcomes were reported for melt blending with MAPP and maleic anhydride-grafted styrene–ethylene/butylene–styrene (SEBS-g-MA), in which the filler had first been treated with NaOH, silane, or a sequential NaOH/HCl/silane procedure to reinforce the action of the coupling agents [17]. Taken together, these results indicate that compatibiliser performance cannot be judged in isolation from fibre pretreatment: it is the combination of the two that determines interfacial quality.

#### 2.1.4. Rice Straw: Fiber Content, Size and Modification

The mechanical properties of rice straw (RS) fibre are the main rationale for its use as a PP reinforcement. RS–PP composites are typically produced by injection moulding, whereby the fibre-laden matrix melt is injected into a mould and subsequently cooled. Filler content exerts a markedly non-monotonic influence on properties. Within 5–30 wt.%, tensile and flexural strength rise up to approximately 20 wt.% and then decline as weak interfacial adhesion takes over; at high loadings, fibre ends act as crack-initiation sites and impair impact resistance. Other researchers, by contrast, observed strength losses even at small RS additions, which they attributed to an inadequate interface; the same studies also examined how fibre dimensions affect tensile strength at break and thermal conductivity [20]. This divergence most likely reflects differences in straw origin and preparation that were not described in sufficient detail to permit a valid comparison.

Given the poor adhesion of RS to PP, research has focused largely on fibre modification. Raising the NaOH concentration and extending the alkaline treatment time increased tensile and flexural strength as well as flexural modulus—presumably because a larger fibre surface came into contact with the matrix—whereas tensile modulus tended to fall [20]. Sequential NaOH and H_2_O_2_ treatment of cellulose isolated from RS lowered the water absorption of the composites compared with their untreated counterparts [21]. Steam treatment of RS decreased the storage and loss moduli of the composite, an effect the authors ascribed to better dispersion and interfacial interaction [22]. Across all loadings, these composites exhibited higher strength, higher Izod impact resistance, and improved thermal characteristics.

In RS/PP, both fibre treatment and MAPP are able to strengthen interfacial interaction [20]. The optimal loading, however, is formulation-specific. In study [21], incorporating 20 wt.% rice straw cellulose increased Young’s modulus from 638.5 to 731.3 MPa but reduced strength from 34.3 to 28.1 MPa and elongation from 57.5 to 10.9%. A loading of 20 wt.% therefore cannot be regarded as a universal optimum for packaging.

RH/PP likewise calls for fractionation, drying, and surface treatment to be coordinated. Chemical pretreatment may improve adhesion while simultaneously raising reagent consumption and cost, so its environmental burden must be weighed alongside the resulting composite properties [6,15,16,23,24].

The appropriate loading depends on the intended application: rigid containers require stiffness with preserved impact resistance, whereas films additionally demand drawability, puncture resistance, and seal integrity. Comparing fillers across different matrices and test methods does not reveal a general optimum.

### 2.2. Biogenic CaCO_3_ from Waste (Eggshells and Mollusk Shells)

Calcium carbonate recovered from eggshells and mollusc shells (bio-CaCO_3_) has developed into a distinct line of materials research aligned with circular-economy principles. Two factors drive this interest: the opportunity to valorise substantial biowaste streams and the prospect of substituting ground natural CaCO_3_ in polymer matrices. Because waste availability varies by region, it must be assessed locally [25]. Within PP, micronised eggshell calcite functions as an accessible, low-cost filler that increases stiffness and thermal stability; provided that the particle-size distribution is optimised and the surface is treated with a coupling agent, strength can be maintained at a level adequate for rigid containers and lids.

Ground limestone has long served polymer processors as an inexpensive filler that lowers cost and adjusts material properties. Shells of hen eggs and of marine molluscs—mussels, oysters, scallops, cockles, and others—arise during egg and seafood processing, contain CaCO_3_ in amounts comparable to this sedimentary rock, and could in principle replace it. At present, only a small fraction finds use in feed supplements, liming of acidic soils, or wastewater treatment, while the bulk is sent to landfill [26]. As they decompose, these wastes emit pungent ammonia, hydrogen sulfide, and amines, attract rodents and insects, and act as reservoirs of pathogens [27]. Industrial efforts to valorise them have already reached the pilot stage. In the United States, American Dehydrated Foods is working with the Georgia Institute of Technology on a facility that recovers CaCO_3_ from eggshells for use in plastics, partially displacing petrochemical feedstock; in the United Kingdom, Just Egg and the University of Leicester have set up a pilot line producing shell powders as reinforcing fillers [28].

#### 2.2.1. Sources of Biogenic CaCO_3_ and Their Characteristics

Naturally occurring CaCO_3_ exists as calcite, aragonite, and vaterite, and its density and morphology affect both the volume fraction and the distribution of the filler [29,30]. Any comparison between biogenic and mineral CaCO_3_ must therefore be made at matched particle size, surface treatment, loading, and PP grade.

In a hen’s egg, about 60% of the mass is albumen and about 30% yolk, with the shell and its inner organic membrane making up the remaining 10–11%. The shell consists of 94–97% CaCO_3_ in calcite form [31,32], 3–4.5% organic matter [33,34], and trace amounts of other elements; it is easily ground into micro- and submicron fractions and is generated in large volumes as a food-industry by-product. Shells of marine molluscs—mussels, oysters, scallops, snails, and cockles—contain 92–99% CaCO_3_ [35] and roughly 5% organic matter [36]. Both sources thus match natural limestone in purity, but their organic fraction is larger and more variable, which has direct consequences for filler preparation.

#### 2.2.2. Physicochemical Characteristics of Bio-CaCO_3_ Relevant to Composites

Reinforcement by bio-CaCO_3_ may depend on the polymorph and particle morphology [37,38,39]. Their individual contribution to PP properties, however, cannot be isolated when purification, particle size, and surface treatment are varied at the same time; a causal comparison requires a single matrix and harmonised processing conditions.

A second decisive parameter is particle size and its distribution. Micron-sized powders consistently raise modulus and stiffness—and, in some systems, wear and scratch resistance—but become stress concentrators at high concentrations. Owing to their large specific surface area, nano- and submicron fractions can, in principle, transfer stress more efficiently, provided that dispersion and interfacial interaction are tightly controlled. A recent review of shell- and eggshell-derived bio-CaCO_3_ treated this duality systematically: reinforcement when dispersion is good, degradation of properties when particles agglomerate [40]. Practical confirmation comes from mussel-shell filler in PP. Controlled dispersion could not be achieved without surface modification, whereas a polydopamine coating, MAPP, and especially both together improved particle distribution and adhesion to the matrix, making it possible to exploit the advantages of fine fractions without increasing defect density [41].

The residual organic (including biopolymeric) phase on particle surfaces deserves separate consideration. Although it occasionally aids interfacial compatibility, it far more often widens the scatter of properties because of contaminants, retained moisture, and a tendency to agglomerate. Current studies therefore make extensive use of washing, chemical purification, heat treatment, and calcination, followed by surface functionalisation with stearate or other organic layers. For shell-derived bio-CaCO_3_, a stearate coating proved to be a practical route to better processability and filler quality, although the thickness and chemistry of the organic layer must be controlled [42]. For eggshell, calcination was shown to improve interfacial bonding relative to the untreated material [43]. The underlying principle is that the extent to which the organic phase is removed or transformed governs the quality of the interface and, in turn, the mechanical behaviour of the composite.

From the literature of the past 5–7 years, three practical guidelines for designing biofilled polymer composites can be distilled. First, polymorph content (calcite versus aragonite) is more than a mineralogical descriptor: it can dictate modulus, hardness, and fracture mechanism. Second, moving to nano- and submicron fractions broadens the reinforcement potential only when dispersion and interfacial adhesion are deliberately managed. Third, the organic phase of shells should be treated as a process parameter that is stabilised through purification, heat treatment, or controlled surface modification. Datasets in which these three factors are varied independently within one PP matrix, however, are virtually non-existent.

#### 2.2.3. Preparation and Modification Methods for Biogenic CaCO_3_

There is broad agreement in recent work that untreated bio-CaCO_3_ particles deliver only modest mechanical gains: stiffness and modulus increase, yet without strong interfacial adhesion, strength and impact resistance improve marginally or even deteriorate. Review [40] attributes this directly to agglomeration, a weak interface, and variability of the particle surface, and identifies surface modification as among the most dependable means of improving compatibility. Experiments with mussel-shell filler in PP point to the same conclusion, since dispersion and adhesion remained the limiting factors in the absence of modification [41]. The preparation methods are discussed below in order of increasing complexity, progressing from mechanical treatment to chemical and hybrid functionalisation.

Mechanical comminution and micronisation form the basic preparation step. Ball milling and other high-energy techniques reduce particle size, alter particle shape, and narrow the size distribution, thereby improving the reproducibility of the reinforcing effect. Micronisation alone, however, does not guarantee better properties, because finer particles bring a larger specific surface area and a stronger tendency to agglomerate. Studies on micronised shell particles in organic coatings report higher wear resistance and modulus while interfacial defects persist [44].

Heat treatment and purification aim to eliminate residual organics, moisture, and surface contaminants. This step matters especially for bio-CaCO_3_, whose organic phase may either take part in or obstruct interfacial interaction. Beyond cleaning the surface, heating triggers phase transformations—above all the conversion of aragonite to calcite and, at still higher temperatures, decarbonisation to CaO. Heat treatment should therefore be viewed as a tool for controlling the crystalline state and mechanical potential of the filler, not merely as a cleaning operation [45,46,47].

Stearate treatment remains the most widespread chemical strategy for hydrophobic thermoplastics. By lowering the surface energy of CaCO_3_, stearic acid and its salts suppress agglomeration and enhance compatibility with non-polar matrices such as PP and PE. Formation of a stearate layer on shell-derived bio-CaCO_3_ has been verified experimentally and is regarded as a practical way to improve processability and interfacial compatibility. Review data nonetheless emphasise that the effect depends on dosage and coating uniformity and that an excess of modifier is undesirable [42,48].

Silane coupling agents are widely employed for polar matrices, including epoxy resins, polyamides, and other systems in which interfacial chemistry is critical. Silanisation is considered one of the principal means of enhancing adhesion and limiting agglomeration of bio-CaCO_3_, and experiments with marine mollusc shells confirm better particle distribution and matrix contact under targeted functionalisation. Although the specific silane is chosen according to the polymer chemistry, the general mechanism is that the silane acts not as a passive coating but as a molecular bridge linking the inorganic surface to the organic phase [49].

Polymeric coatings and surfactants constitute a further group of agents that improve dispersion and reduce particle sticking and filler abrasiveness. In the mussel-shell/PP study cited above, the best dispersion and adhesion were obtained precisely when a polydopamine coating was combined with MAPP, which is especially important for fine fractions with high surface energy [41]. Reviews of nano-CaCO_3_ modification likewise single out polymeric coatings as a promising class of surface engineering capable of simultaneously improving compatibility, limiting agglomeration, and preserving processability [49]. The newest direction involves hybrid and nanocomposite approaches in which CaCO_3_ serves not only as a mechanical filler but also as a carrier for an additional function: metals or their oxides are deposited onto the particles to impart antibacterial, catalytic, or photocatalytic properties [50]. While such hybrids hold promise for active PP-based food packaging, questions regarding their migration and safety remain unresolved.

### 2.3. Eggshell Fillers in PP

Eggshell-derived biogenic CaCO_3_ is considered a partial substitute for mineral fillers in PP. Data for other thermoplastics and thermosets are not used here to select PP loadings, because wetting, processing, and stress transfer differ between these matrices [25].

Even without modification, eggshell markedly increases the Young’s modulus of PP, particularly at high loadings. At 40 wt.% of particles below 38 μm, the modulus rose by approximately 136% relative to unfilled PP, and with a broader 14–157 μm distribution at the same loading the increase reached 220% [25]. This behaviour, typical of mineral fillers, stems from the introduction of rigid, non-deformable particles. Tensile strength follows a different course: it is essentially constant at 1–5 wt.% but declines with further filling, ending up 30–40% below that of the neat polymer at 30–40 wt.% [25]. The decline is attributed to incompatibility between the hydrophobic PP matrix and the hydrophilic particle surface, to agglomeration, and to the formation of stress concentrators.

Impact resistance is the most contradictory property. Some studies report a 38–48% increase in Charpy impact strength at 3–5 wt.% filler, linked to a change in fracture mechanism and energy dissipation in the vicinity of the particles. In other studies, Izod impact strength decreased at every filler content, pointing to the dominance of structural defects and agglomerates [51]. Because the test methods (Charpy versus Izod), particle-size distributions, and processing conditions all differ, the contributions of these factors cannot be separated unambiguously; one can only note the high sensitivity of impact behaviour to particle size, dispersion quality, and processing conditions.

Eggshell must be compared with commercial CaCO_3_ at equal fineness and with identical surface treatment. Poly(propylene carbonate) (PPC) differs chemically from polypropylene, so results obtained for PPC do not characterise PP packaging [52].

In short, eggshell can stiffen PP, but whether strength and impact resistance are retained depends on dispersion, interfacial adhesion, and the processing regime.

### 2.4. Mollusk-Shell Fillers in PP

PP remains the principal thermoplastic host for mollusc shell (SS) fillers, which serve to increase stiffness while strength changes in a formulation-dependent manner. In one study, nanodisperse SS powder below 200 nm was obtained by washing, alkaline treatment with 4% NaOH, and fine grinding; it was then compounded in a twin-screw extruder and injection-moulded. Young’s modulus peaked at 10 wt.% (+85%), whereas the strength optimum corresponded to loadings of up to 5 wt.%, with strength falling by about 13% [25]. Relative to commercial CaCO_3_ of comparable fineness (40–500 nm), the PP/SS composites displayed a more balanced property profile, which the authors attribute to the residual organic phase of SS presumably strengthening the filler–matrix bond.

Micron-sized fractions of 2–4 μm at 5–30 wt.% exhibit the characteristic trade-off between stiffness and strength. Tensile strength falls monotonically by 3–21%, while flexural modulus rises non-linearly and exceeds the reference value by 62% at 30 wt.%. Charpy impact energy grows by 5–17% for all formulations, peaking at about 10 wt.% [25]. The strength loss is linked to weak adhesion, weakened interfacial zones, and a smaller proportion of continuous polymer phase, whereas the modulus gain reflects a larger volume fraction of rigid phase and embrittlement. Hence, at micron-scale fineness, moderate shell loadings enhance stiffness—and to some extent impact resistance—of PP, whereas higher concentrations compromise strength.

Similar trends are observed for snail shells with particles smaller than 10 μm. Over 5–30 wt.%, the tensile strength of PP decreased by only 0–9%, while Young’s modulus increased almost linearly to +100% at 30 wt.%. At 30 wt.%, mineral CaCO_3_ outperformed the biogenic filler in strength and modulus by only about 3%, indicating satisfactory dispersion and acceptable adhesion of the biofiller under the chosen compounding conditions [53]. These data confirm that, with a suitable particle fraction and mixing technology, shell-derived CaCO_3_ can compete with commercial calcium carbonate as a stiffening agent for PP.

All carbonate fillers share one limitation: hydrophilic CaCO_3_ is incompatible with hydrophobic polymers, necessitating surface modifiers that suppress agglomeration and improve adhesion. An overly thick coating can impair strength, and differential thermogravimetry (DTG) makes it possible to distinguish a monomolecular modifier layer from a multilayer coating [25]. Stearic acid is the most common modifier for CaCO_3_; polyacrylic and acrylic acids, phosphates, silanes, dendritic carboxylic acids, titanates, and zirconates are also used. For eggshell CaCO_3_, modification has been described with propionic acid [54]; with silanes, titanates, and zirconates [55]; with stearic, isophthalic, and pimelic acids; and with maleic anhydride, as well as by solid-state shear milling without chemical treatment. The range of modifiers applied to marine shells is narrower so far: silanes, stearic acid [56], titanates [57], and pimelic acid [58]. Surface modification generally improves mechanical properties, yet no systematic comparison of modifiers within one matrix at the same loading is available.

Taken together, Section 2.3 and Section 2.4 confirm that biogenic CaCO_3_ can be used to tune PP stiffness, but they do not permit sources to be ranked independently of fineness and treatment. Section 8.1. “Concept of Functional Separation” keeps tensile and flexural results separate, and strength and modulus values obtained at different loadings are not treated as paired. The most informative benchmark remains a comparison with mineral CaCO_3_ under harmonised conditions [53].

### 2.5. General Trends in the Use of Agri-Food Waste in PP Composites

Pits, shells, peels, oilseed cakes, and pomaces are incorporated into PP both as functional fillers and as antioxidant carriers. Their performance hinges on three conditions: drying to a stable moisture level, control of fineness with a narrow size distribution, and the use of interfacial compatibilisers—most often MAPP and, less frequently, reactive copolymers. Olive pit flour offers an instructive example: loadings of up to 70 wt.% increase the modulus and stiffness of PP while keeping processability acceptable. Achieving this requires uniform dispersion, dry fractions (typically below 250–500 μm), and intensive heat removal during injection moulding to prevent thermal degradation of the biophase [59].

Spent coffee grounds (SCG) obey the same rules. Early work found that 20 wt.% SCG in PP raises modulus and hardness, whereas impact resistance depends on how completely the coffee oils are removed and on the particle-size distribution; MAPP evened out adhesion and lowered water uptake, and SCG itself behaved as a weak nucleating agent that altered PP crystallinity [60]. In packaging-oriented studies, SCG was heat-treated in air. Removing the oils made the particles easier to grind and better wetted by the matrix, and the resulting composites absorbed less water and showed superior mechanical properties—findings that justify further evaluation for packaging, subject to verification of barrier properties, migration, and food contact [61]. When different thermoplastics were compared at high SCG contents, the combination of drying, fractionation, and compatibiliser gave PP the most predictable processing and the most balanced properties. Polar matrices carry a lower risk of phase incompatibility, but cooling–heating cycles intensify water sorption and brittleness in them [62].

#### 2.5.1. Olive Industry Waste

Data on olive pits illustrate most clearly what a compatibiliser contributes. Pit flour raises the elastic modulus of PP, while excessive filling lowers tensile and flexural strength; adding MAPP substantially increases the strength of such composites [59]. Likewise, in PP blends containing 0–40% olive pit flour and almond shell, the modulus grew with loading, whereas ultimate strength, elongation at break, and impact resistance declined because of weak interfacial bonding [63,64]. With 5–20% pit particles and maleic anhydride, particles remained uniformly distributed up to 15%, but the surface became rough at 20% [65]. Of particular note, at 20% filler the oxidation induction period of PP rose from 1.15 to 30.1 min, and thermogravimetry revealed enhanced thermal stability—evidence that the pits possess intrinsic antioxidant activity [65].

Other feedstocks confirm the role of the compatibiliser. Hybrid composites containing up to 50% coconut fibre and up to 20% coconut shell particles showed a higher PP elastic modulus, with MAPP improving bonding and reducing water absorption [66]. The same patterns hold for olive pits in recycled PP (rPP): the flour increased hardness, modulus, and dimensional stability, while MAPP-based compatibilisers mitigated the loss of impact resistance [67]. The authors particularly stress that low residual moisture in the flour is essential for minimising defects when packaging containers are moulded [67].

#### 2.5.2. Nut Shells

Almond shell is a lignocellulose-rich filler. Related work has also shown that, in bio-PP filled with mango peel flour, an environmentally benign itaconic acid-based compatibiliser can take over the role of MAPP [68]. Combining MAPP with small amounts of montmorillonite reduced interfacial porosity and water absorption, raised the glass transition temperature measured by DMA, and stabilised dimensions during cooling–heating cycles; the authors recommend nanoclay when the bioflour content is high, i.e., at least 30 wt.% [69]. Further studies support this division of labour, in which MAPP provides bonding while dispersed nanoclay slows moisture diffusion; with fractions below 250 μm, impact resistance decreases only moderately [70]. Torrefied almond shell in rPP–PE blends increased heat resistance and modulus and reduced hygroscopicity—results that merit investigation but do not prove suitability for hot-fill packaging [71].

Peanut shell yielded comparable results. In recycled PP containing 0–40% shell powder, the melt viscosity (assessed via torque) and the elastic modulus increased, whereas strength and elongation fell [72]. The compatibiliser poly(ethylene-co-acrylic acid) (PEAA) improved strength and lowered water absorption, and FTIR spectroscopy and SEM confirmed chemical interaction between filler and matrix [72]. Following electron-beam treatment and six months of natural ageing, the irradiated specimens retained higher strength and heat resistance at the cost of reduced elongation; the rise in carbonyl index was accompanied by surface biodegradation [73]. At 50% peanut flour with the organomodified montmorillonite Cloisite 30B, combining maleated PP (PPM) with maleated SEBS (SEBSM) increased both modulus and impact resistance: PPM supplied strength and fracture resistance, while SEBSM contributed elasticity [74].

Shells of hazelnut, walnut, pistachio, coconut, and argan are also used in PP composites [75,76,77,78,79,80]. The common governing factors are fineness, residual moisture, and interfacial adhesion. For packaging use, mechanical data must be supplemented by evaluation of the surface, odour, barrier properties, and migration of the finished article.

#### 2.5.3. Fruit Stones

Extruding apricot-kernel particles with MAPP improved dispersion and the modulus of PP, and SEM revealed fewer interfacial voids [81]. This, however, is not a measurement of gas transport or seal integrity. Fruit stones require control of melt viscosity and thermal history so that PP degradation and overheating of the biophase are avoided [82].

#### 2.5.4. Date Seeds and Oilseed Cakes

Research on date seeds has focused mainly on thermal behaviour. Their blends with PP decomposed in two stages; moisture and the hemicellulose fraction reduced thermal stability relative to unfilled PP, and isoconversional analysis yielded activation energies of 51–156 kJ/mol [83]. Oilseed cakes of black cumin and rapeseed in post-consumer PP produced composites with increased stiffness and acceptable strength, with defatting, drying, and sieving of the cake identified as prerequisites for stable adhesion and low moisture sorption [84].

#### 2.5.5. Grape Processing Waste

Grape residues contain polyphenolic antioxidants and antimicrobial compounds of interest for active packaging [85]. Data obtained directly on PP films are still scarce. Carriers can regulate whether the extract is retained or released, but the presence of a carrier does not in itself demonstrate the absence of migration.

**Figure 2:**
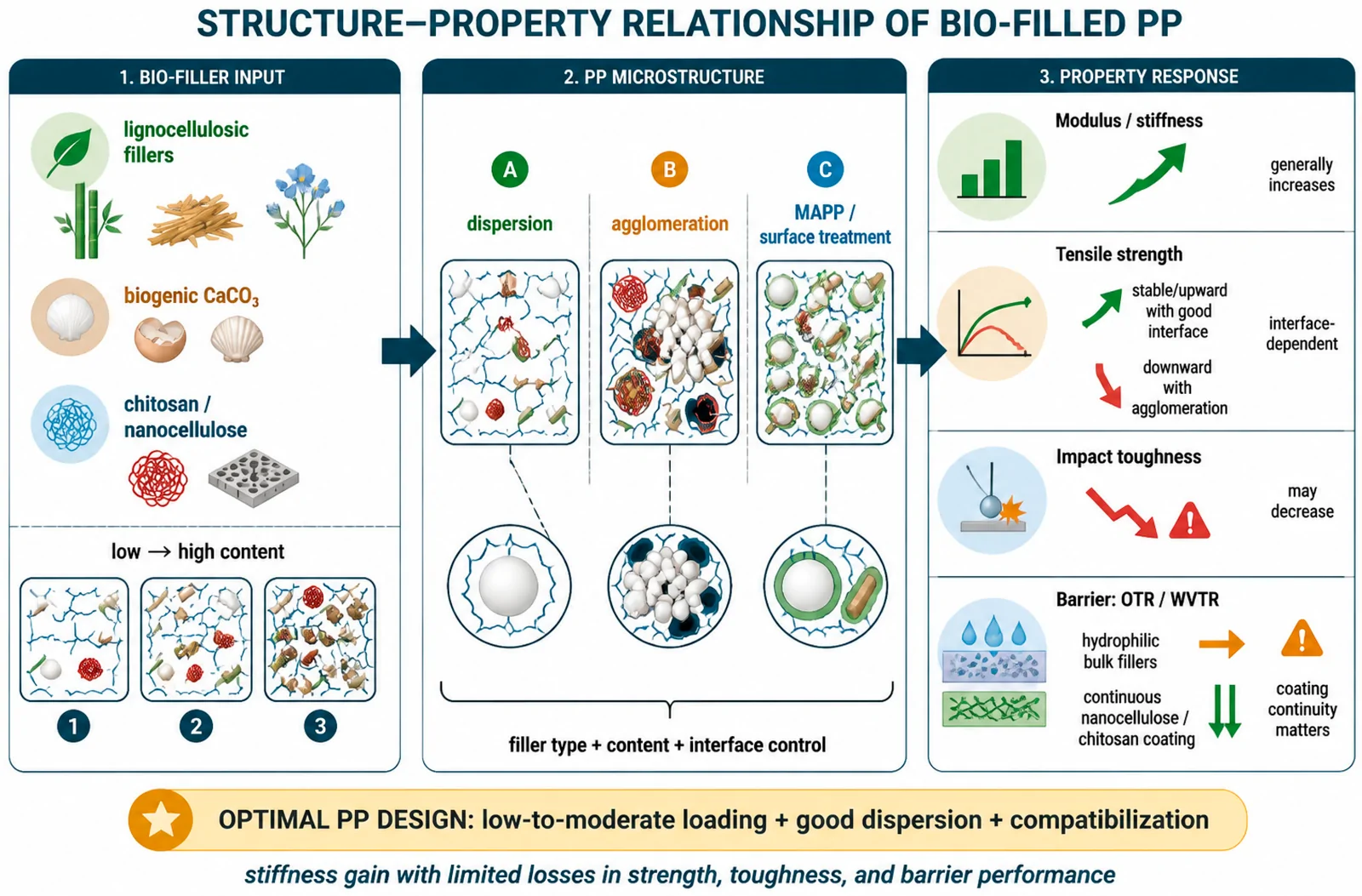
Structure–property relationships in biofilled PP: influence of biofiller type and content and of interfacial control on modulus, strength, impact resistance, and barrier properties. This figure was generated using Grok AI 4.3 and adjusted by the authors to align with the logic presented in the manuscript.

Overall, bulk modification with agricultural waste can raise the modulus and stiffness of PP (Figure 2), yet its effect on barrier properties and carbon footprint is formulation-dependent. The main gap is that mechanics, migration, and barrier are seldom evaluated together on the same finished packaging article.

### 2.6. Effect of Lignocellulosic Fillers on PP Properties

Because of their hydrophilicity and the interfacial voids they create, lignocellulosic particles can increase water absorption at the same time as they raise stiffness [6]. Water absorption, however, is not equivalent to WVTR, and any change in oxygen transport cannot be inferred from hydrophilicity without measurement. Compatibilisers and surface treatments reduce defects, but the barrier effect has to be verified separately.

#### 2.6.1. Role of MAPP in Interfacial Chemistry

By improving wetting and filler–PP adhesion, MAPP can also reduce water absorption [86,87,88]. The 2–4% (relative to matrix mass) cited in the literature is a starting point for formulation rather than a universal optimum. Grafting degree, dosage, and calculation basis should be reported, and barrier and migration characteristics should be verified independently.

#### 2.6.2. Barrier Properties and Surface Protection

Thin coatings offer a means of controlling oxygen transport without assigning every function to the bulk filler [89,90]. For hydrophilic nanocellulose and PVOH layers, the influence of humidity, as well as layer continuity, adhesion, and damage during forming, must be checked. A reduction in carbon footprint should not be assumed automatically.

#### 2.6.3. Bast Fibers

Hemp fibre stiffens PP, while MAPP strengthens interfacial interaction and lowers water absorption [91,92]. In packaging, fibre length must be matched to wall thickness, and a low fibre fraction does not by itself ensure that the barrier is preserved.

#### 2.6.4. Environmental Performance of Bulk Modification

Life-cycle assessments of biocomposites show that outcomes depend on formulation, system boundaries, transport, drying, and the allocation of waste-related burdens [93,94,95]. Substituting virgin PP therefore does not establish an environmental advantage unless functionally equivalent packages are compared; no universally environmentally optimal loading range has been identified (Table 2).

## 3. Waterborne Coatings and Primers on PP

Whether the aim is a barrier or active coating or a primer for printing and bonding, a waterborne layer cannot be applied to PP until its low-energy surface has been activated [96]. Corona discharge is the routine treatment in PP film converting, whereas plasma activation is chosen when deeper or more uniform modification is needed [97]. The quality of the activated layer largely determines the adhesion and stability of the entire multilayer structure.

### 3.1. Nanocellulose-Based Waterborne Barrier Coatings

Cellulose, first described by Anselme Payen in 1838, is the most abundant biopolymer on Earth and is synthesised mainly by plants and by certain bacteria, among them *Acetobacter xylinum* [98]. Cellulose nanocrystals (CNC) are produced by acid or oxidative hydrolysis, during which the amorphous regions are degraded faster than the crystalline ones, liberating highly ordered crystalline domains [99]. A dense network of surface hydroxyl groups renders nanocellulose (NC) hydrophilic and hygroscopic; this same feature is also its main drawback, causing aggregation and poor compatibility with non-polar solvents and hydrophobic polymers, notably polyolefins [100]. At the same time, this surface chemistry offers ample scope for functionalisation [101]. Modifying compounds are anchored to CNC through physical adsorption or electrostatic interaction with surfactants and cationic groups [102], thereby improving dispersibility, matrix compatibility, and barrier performance against gases and water [103].

Despite promising mechanical and oxygen-barrier properties, nanocellulose is sensitive to moisture and only marginally heat-sealable [104]. On PP films, nanocellulose and nanochitin can strengthen the oxygen barrier at low humidity, with corona treatment and primers improving adhesion [105]. A biological origin, however, does not substitute for assessment of purity, residual reagents, and migration.

Nanocellulose coatings are also being explored as carriers for active components [106,107,108]. For a BOPP/LDPE system bearing a cellulose coating, the OTR fell from 67.03 to 24.02 cm^3^·m^−2^·day^−1^ at 23 °C and 50% RH [109], a reduction of 64.2%. This is a within-study comparison on a mixed polyolefin substrate and does not prove that the same effect would be obtained in mono-material PP. Microbiological results should be compared only when identical methods and test objects are used.

Hydrophobic layers can shield a nanocellulose barrier from humidity, but the suitability of such a structure depends on adhesion and on property retention in service. A biodegradable coating does not make packaging based on conventional PP compostable, and the recyclability of the complete structure must be verified.

### 3.2. Chitosan Coatings

Applied to PP that has been pre-activated by corona or plasma treatment, chitosan obtained from aquatic bioresource processing waste imparts antimicrobial properties to the surface and partially lowers vapour permeability [110]. This approach fits the broader concept of edible films and coatings, which is regarded as one of the cost-effective strategies for maintaining food safety and freshness. Coatings are applied to the product in liquid form, whereas films are formed separately; in both cases the goal is to enhance safety, quality, and shelf life [111]. Active packaging encompasses moisture and gas scavengers, gas emitters, antioxidants, and antimicrobial agents along with their release systems, and is designed to suppress microbial, enzymatic, and oxidative spoilage while preserving colour and nutritional value. Interest in natural active components is growing against a background of critical scrutiny of metal and metal-compound nanoparticles (CuO, Ag, ZnO, Fe_3_O_4_, TiO_2_), which have raised concerns about potential cytotoxicity, genotoxicity, and pro-inflammatory effects, as well as about migration into food and accumulation in the body and the environment [110].

Alginates, pectins, starches, and chitosan are considered versatile active-packaging matrices that combine biocompatibility, low toxicity, and economy with the capacity to carry other active components [110]. When applied to a single-layer plastic film, they can modify its barrier properties, but the resulting multicomponent construction requires separate assessment of recyclability. Among these, chitosan—sourced from crustacean exoskeletons and fungal cell walls—stands out for its good film-forming ability. Its limitations relate to pH-dependent solubility arising from protonation of the amino groups and to reduced antimicrobial efficacy under certain conditions, which has spurred the development of modified and hybrid formulations [112]. Extracts of grape seeds and pomace, pomegranate seeds, blackberry, and juniper; essential oils of thyme, clove, and oregano; and individual phenolics such as caffeic acid, catechin, catechol, and resveratrol have all been investigated in combination with chitosan [110]. Chitosan–phenolic systems have already been incorporated into conventional packaging films, including PP-based ones, although the space of possible combinations is far from fully explored.

Chitosan may be used either as a free-standing film or as a coating. Surface activation demonstrated for PLA/PBS [97] supports the general mechanism of adhesion enhancement, yet the quantitative results cannot be transferred to PP without testing in that matrix.

Edible chitosan films are transparent and possess adequate mechanical strength along with selective permeability to CO_2_ and O_2_ [113]. Their main shortcoming, high water-vapour permeability, restricts their use for moisture-sensitive foods, and chitosan is therefore blended with other hydrocolloids to offset it [113]. It is precisely this limitation that justifies placing chitosan on a hydrophobic PP substrate, which then acts as the moisture barrier.

### 3.3. Waterborne Multilayer and Combined Systems

PP films (BOPP, CPP) rank among the leading flexible-packaging materials thanks to their combination of strength, chemical resistance, low density, and processability, but their functionality is held back by low surface energy and weak adhesion to coatings. Aqueous polymer dispersions allow the surface to be modified without organic solvents, in line with current environmental requirements for manufacturing [96]. The literature on dispersion barrier coatings confirms their industrial applicability when the surface is corona-treated, drying is carried out in several stages, and temperature and humidity—which govern the stability of the oxygen barrier—are controlled. The key packaging metrics, oxygen transmission rate (OTR) and water-vapour transmission rate (WVTR), depend on polymer chemistry, crystallinity, chain packing density, and coating structure [89].

**Figure 3:**
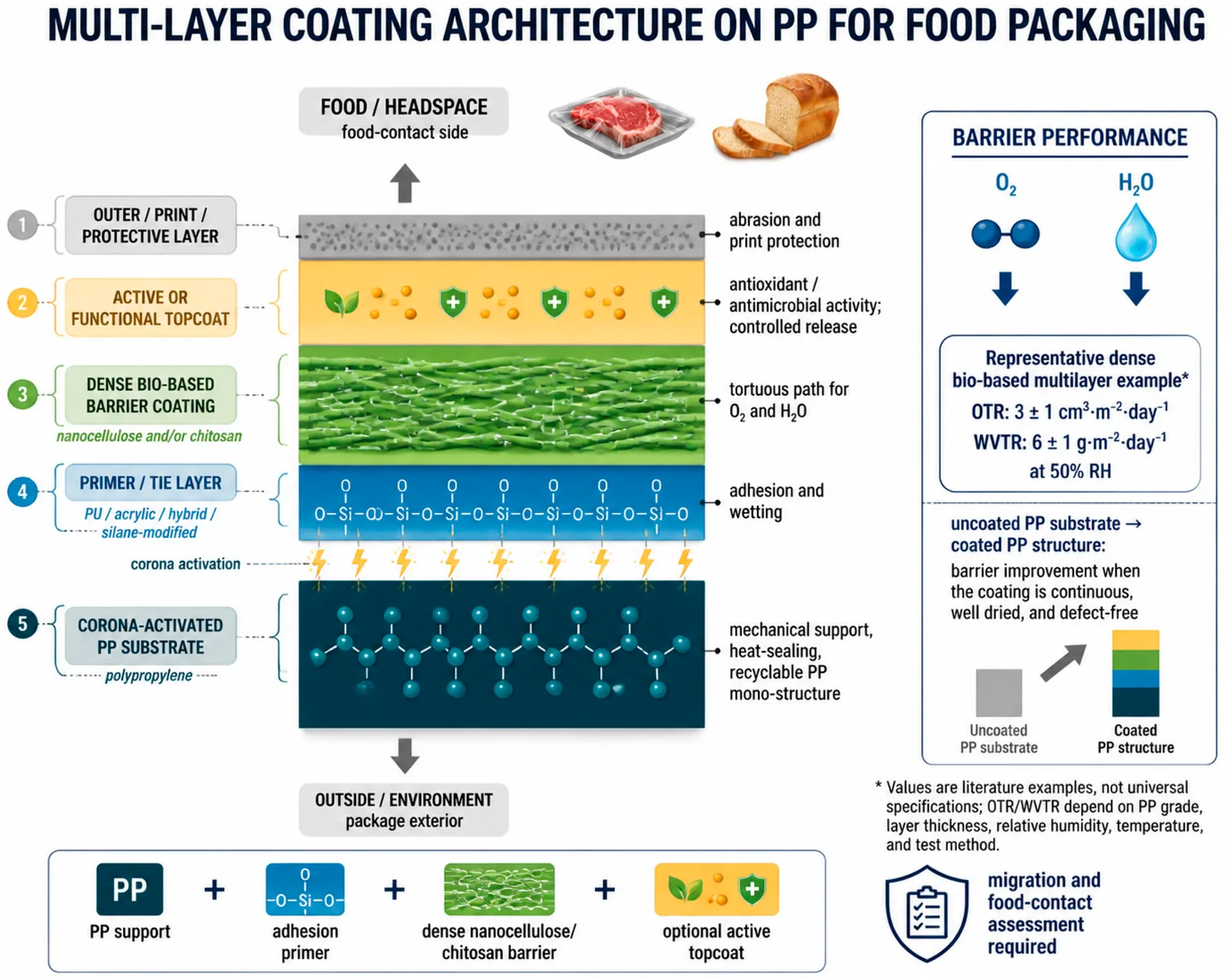
Multilayer coating architecture on a PP substrate, indicating the functions of the layers and representative OTR/WVTR values. The OTR value of 3 ± 1 and WVTR value of 6 ± 1 shown in the scheme are illustrative only: their original source, the layer thicknesses, and the measurement conditions have not been confirmed in this review, and these values are not used in any quantitative comparison of materials. This figure was generated using Grok AI 4.3 and adjusted by the authors to align with the logic presented in the manuscript.

Multilayer coatings make it possible to distribute functions among layers and to combine the attributes of different materials [114]. A typical architecture comprises a PP substrate, an activated surface layer, a primer, a barrier layer, and a protective functional layer (Table 3, Figure 3). The primer bridges the hydrophobic PP surface and the polar barrier coating; the barrier layer provides the main resistance to gas and moisture diffusion; and the protective layer confers resistance to mechanical and chemical attack. By lengthening the diffusion path of gas molecules, this architecture can reduce permeability considerably compared with single-layer coatings [115].

#### 3.3.1. Waterborne Primer Systems

Because the adhesion of every subsequent functional layer to the polymer substrate depends on it, the primer is a key element of any multilayer system. The most widespread types are waterborne polyurethane dispersions (WPU), acrylic latexes, polyurethane–acrylic hybrids, and silane-modified polymers (Table 4) [96]. Primer type strongly affects coating adhesion and performance, and each class strikes its own balance of advantages and limitations.

The highest adhesion is achieved with WPU primers. Their combination of soft and hard segments confers elasticity, good film formation, and resistance to mechanical loading, while their polar groups interact effectively with the activated PP surface. WPU is consequently used extensively in BOPP printing and lamination despite costing more than other waterborne primers. Acrylic dispersions, valued for transparency, UV resistance, and relatively low cost, are common binders for printed and decorative layers; their adhesion to PP, however, is usually inferior to that of polyurethanes, which limits their use in heavily loaded multilayer structures.

Polyurethane–acrylic hybrids unite the strengths of both classes: the polyurethane segments deliver adhesion and flexibility, while the acrylic segments improve resistance to ageing, UV radiation, and chemical attack—hence their growing use in multilayer barrier coatings. Silane-modified primers carry functional groups capable of bonding chemically both to inorganic coating components and to the activated polymer surface, enhancing adhesion and durability, although their efficiency depends heavily on application conditions and ambient humidity. On the whole, polyurethane and hybrid dispersions are regarded as the most effective primers for PP films, with the final choice dictated by requirements for adhesion, barrier, durability, and cost [96].

#### 3.3.2. Barrier Coatings

PVOH and dense hybrid coatings can lower oxygen transmission, but how well they do so depends on humidity, structure, and layer continuity [115,116]. Table 5 lists specific comparisons against control samples, keeping OTR separate from permeance normalised to the pressure difference.

Application of the semi-industrial CBS004 coating to CPP70 reduced oxygen permeance from 1516 to 36 cm^2^·m^−2^·day^−1^·bar^−1^ [115], i.e., by 97.6%. This result cannot be combined with the OTR of system [109] unless pressure normalisation, substrate thickness, and test conditions are reconciled, and no cross-study ranking of coatings is therefore attempted.

Barrier performance is governed not only by chemical composition but also by the absence of pores, cracks, and delamination: the primer secures adhesion, whereas the continuous functional layer provides resistance to transport. Compatibility of coated PP with mechanical recycling must be verified for the structure as a whole, including binders and active additives.

To ensure comparability, the following should be reported: substrate; film and dry-coating thickness; temperature; RH on both sides; pressure difference; method; standard edition; and uncertainty. ASTM D3985 applies to coulometric OTR measurement, ASTM F1927 to measurement under controlled humidity, and ASTM F1249 to WVTR [117,118,119]. These standards are not retrospectively attributed to studies that do not describe their methodology, and wet application thickness must not be substituted for dry-layer thickness.

### 3.4. Active PP Packaging Based on Natural Extracts

In active packaging, the material itself performs an additional protective role by preventing oxidation, spoilage, or microbial growth. Essential oils, phenolic extracts, nisin, and other natural agents can be introduced either into a coating or into the PP bulk—including on an agro-filler carrier—but release kinetics and migration must be controlled in both cases. The shift in interest from synthetic preservatives to natural extracts reflects concerns about the long-term effects of prolonged consumption of synthetic additives [120,121]. Essential oils and phenolic extracts exhibit pronounced antioxidant and antimicrobial activity; polymers containing them retain their core properties while gaining a shelf-life-extending function [122]. Reviews cover a wide range of natural additives—from essential oils to pigments and peptides—together with their mechanisms of action and the factors governing their efficacy [123]. Their technological drawbacks, namely high volatility, low solubility, and instability [124], define the central challenge: delivering the component to the food in a controlled manner without unduly affecting odour and taste [125,126].

#### 3.4.1. Natural Active Agents and Their Modes of Action

Plant essential oils (EO) and phenolic extracts are the most widely used agents. Essential oils are volatile aromatic liquids composed chiefly of terpene hydrocarbons and their oxygenated derivatives, such as limonene, pinene, eugenol, cinnamaldehyde, linalool, thymol, and carvacrol [121,127]. Thyme, clove, cinnamon, and oregano oils are rich in phenolic constituents with strong antimicrobial and antioxidant action [128]. Extracts of rosemary, green tea, and grape and olive pomace, which contain polyphenols, flavonoids, organic acids, tannins, and vitamins, are also employed [129,130,131,132,133,134]. Many of these extracts are obtained from agro-industrial by-products, consistent with the concept of zero-waste production [135,136]. Grape pomace and moringa leaf extracts enhanced the antioxidant activity of packaging and slowed lipid oxidation during meat storage, while olive pomace extract in a PLA/PP blend inhibited bacterial and mould growth on fresh-cut fruit [135].

Nisin forms a separate group: an antimicrobial peptide (bacteriocin) produced by *Lactococcus lactis*, it has long been used as a food preservative against *Listeria* and other Gram-positive bacteria. A 2024 review ranked nisin among the most promising active-packaging agents, alongside phenol-rich extracts of citrus peel, watermelon rind, and noni leaves [128]. Natural agents thus span volatile essential oils, non-volatile extracts, and antimicrobial proteins, all sharing the aim of suppressing undesirable microflora and oxidative processes.

These agents act through different mechanisms. Phenolic components—thymol, carvacrol, eugenol—insert into bacterial lipid membranes, disrupt their integrity, and cause leakage of cell contents, besides inhibiting enzymes and denaturing proteins. Certain terpene aldehydes and ketones penetrate the cell and, according to available evidence, may interfere with DNA replication. Antioxidant action derives from phenolic hydroxyl groups, which scavenge free radicals and terminate lipid-oxidation chain reactions. The multicomponent nature of extracts often gives rise to synergy, with terpene hydrocarbons—weakly active on their own—facilitating the access of phenolics to their targets or stabilising their active form [122,124,137]. Consequently, active packaging containing natural additives can simultaneously retard microbial spoilage and oxidative changes in the product, such as rancidity and browning.

#### 3.4.2. Incorporation Routes: Coating Versus Bulk Modification

Owing to the volatility of essential oils and many extracts, these agents migrate rapidly out of the polymer. Direct incorporation of EO into a plastic usually leads to uncontrolled release of most of the substance early in storage, known as burst release [125]. The result is excessive odour and off-flavour and, more importantly, an agent concentration that falls below the effective level by the end of storage. Effective active packaging therefore needs gradual, sustained release that maintains activity throughout the shelf life [126].

Diffusion can be slowed by micro- and nanoencapsulation, nanocomposites, and other means of binding the active agent. Embedding EO in a nanostructured matrix lengthens the diffusion path: turmeric essential oil enclosed in magnetic silica nanoparticles within a chitosan film, for example, was released gradually and extended the shelf life of surimi [125]. Release kinetics can likewise be tuned by liposomes, nanosomes, emulsions, biopolymer conjugates, and adsorption on porous particles, and nanoclays, nanocellulose, and cyclodextrins retain oil molecules and release them slowly [138,139]. Hydrophilic polymer carriers such as chitosan, starch, and PVA further retard release through hydrogen bonding with phenols and esters [140]. Active films containing natural extracts on polymer carriers extended the shelf life of salami, sweet pepper, minced meat, and bread [141,142,143,144], and PLA–chitosan formulations with rosemary extract are regarded as a basis for such materials [145]. None of these studies, however, made a direct comparison with PP containing freely incorporated oil.

#### 3.4.3. Release Kinetics and Determinants of Efficacy

Four groups of factors determine how fast an active additive migrates [146]. The first is the nature of the agent: small, volatile EO constituents migrate quickly, whereas large polyphenols and peptides, including nisin, move far more slowly. The second concerns the properties of the PP matrix—crystallinity, porosity, plasticisers, and compatibility with the agent. Hydrophobic PP retains non-polar molecules but offers little resistance to the escape of highly volatile compounds, whereas nanoclays and other modifiers slow release by creating a tortuous diffusion path. The third is package thickness and structure: an agent escapes faster from a monolayer film than from a multilayer film with an internal active layer. The fourth comprises storage conditions; higher temperature accelerates diffusion and evaporation, and high humidity may plasticise the polymer or dissolve the coating—an effect sometimes exploited as a release trigger [146]. Microcapsules and nanoemulsions provide more uniform distribution and slower release, and Pickering emulsions stabilised by solid nanoparticles prevent oil coalescence and enlarge the oil–matrix contact area [138,139,147].

Five main strategies are used to regulate release kinetics. Immobilisation, i.e., chemical anchoring of active molecules to the polymer, seeks to limit migration and to sustain surface action without transfer into food [146]. Encapsulation in gelatine, starch, or lipid microcapsules, nanoemulsions, and cyclodextrin complexes within a PP composite affords gradual release and protection from degradation. Porous carriers serve as reservoirs: attapulgite loaded with garlic oil releases it more slowly than a polymer containing freely incorporated oil [148], and zeolites, silica, and plant fibres fulfil a similar role while also protecting the agent from evaporation during processing. In multilayer structures, the active layer is sandwiched between layers of unfilled polymer so that the outer layer limits losses to the environment and the inner, food-contact layer regulates delivery. Finally, stimuli-responsive systems intensify release in response to rising humidity, for instance condensate formation [149], or to changes in pH.

For volatile agents, post-extrusion coatings may be preferable to incorporation into the hot PP melt. The protective effect of the carrier, retention of the agent, and release kinetics all need to be measured [148]. An intermediate PP layer does not guarantee a functional barrier against volatile substances. Intended release and unintended migration are assessed separately, in accordance with the requirements for active materials [150,151].

Antimicrobial efficacy should be expressed as Δlog_10_N = log_10_N_control_ − log_10_N_treated_ at identical times and conditions, specifying the strain, initial inoculum, food matrix, and units of CFU/g or CFU/cm^2^. Converting a 94% inhibition into 1.22 log_10_ is an arithmetic example that holds only for comparable viable-cell counts, and whether the original method satisfies this condition has not been confirmed. For an active layer, release over time, storage temperature, and the time to reach the spoilage criterion are additionally required, since in vitro activity cannot replace product testing. A quantitative shelf-life extension is not established here because verified data from the relevant food trial are lacking [109].

## 4. Barrier Properties and Architectures

Traditionally, the modest intrinsic oxygen barrier of PP has been compensated for by multilayer structures containing EVOH or by metallisation. Since recyclability depends on the structure as a whole, the central task has shifted toward mono-material PP packaging whose barrier is built from micro- and nanofillers and/or waterborne coatings [115,152]. Within layers and coatings, nanoclay and nanocellulose can lower OTR many times over. In a filled PP matrix, however, this barrier benefit is lost when micropores are present, when the particles are poorly wetted by the matrix, or when the filler absorbs substantial water. Among the additives under study for modifying PP—including enhancement of its oxygen barrier—graphene and its derivatives [153,154] stand out, as do the natural materials nanocellulose [155] and nanoclay [156,157].

Being inexpensive and abundant, nanoclay is an attractive natural material for PP nanocomposites [152]. Montmorillonite (MMT), a member of the smectite group, has a 2:1 layered structure in which an octahedral alumina sheet is sandwiched between two tetrahedral silica sheets [158]. The individual platelets, about 1 nm thick and several hundred nanometres long, have a high aspect ratio but are initially assembled into stacks. Delaminating these stacks and distributing the platelets uniformly in the PP matrix creates a tortuous diffusion path that impedes gas transport. Polymer–nanoclay composites are prepared by solution casting, including with biomimetic materials [159], in situ polymerisation [158], layer-by-layer deposition [160], and melt blending [161]. Melt blending is the most practical route for non-polar PP, yet it is precisely here that full exfoliation without organic modification of the clay and a compatibiliser remains an unsolved problem.

## 5. Migration, Safety and Regulatory Requirements

In the EU, food safety rests on Regulation (EC) No 178/2002 [162], which lays down the general principles of food law and establishes the European Food Safety Authority (EFSA), the body responsible for scientific risk assessment, including that of food-contact materials (FCM). Every material and article intended to come into contact with food must comply with framework Regulation (EC) No 1935/2004 [163] and with the good manufacturing practice requirements of Regulation (EC) No 2023/2006 [164]. The framework regulation prohibits transfer of material constituents into food in quantities that could endanger human health or bring about an unacceptable change in the composition or organoleptic properties of the food. It also governs active and intelligent materials, authorisation of new substances, labelling, declarations of compliance, and traceability. In addition, specific measures apply to individual material groups, including plastics and ceramics, and national rules cover non-harmonised materials.

Plastic food-contact materials fall under Regulation (EU) No 10/2011 [150]. Compliance is determined by the composition, substance restrictions, and intended use of the finished article. An overall migration test does not replace the evaluation of individual migrants and NIAS. Rather than providing a general list of packaging polymers, Table 6 sets out the risks of waste-derived components that bear directly on PP.

Food simulants and test conditions are selected according to Annexes III and V of Regulation No 10/2011 for the specific food and conditions of use [150]. Simulant A is 10% ethanol (*v*/*v*), simulant B is 3% acetic acid (*w*/*v*), and D2 is a vegetable oil of regulatory specification; D1, 50% ethanol, is not a universal substitute for D2. Contact time, temperature, and surface-to-volume ratio are chosen to reflect the application, and 10 days at 40 °C is not a universal regime. Testing is carried out on the finished article after its complete thermal history, coating, and ageing.

Active components may function either on contact or through release. Authorisation of a substance as a food additive does not prove that the packaging system as a whole is safe. The dose of active agent, unintended migrants, and organoleptic impact under the foreseen use must be measured separately [148,151,167].

Low-molecular-weight migrants, generally below 1000 Da, receive particular attention because they can be absorbed in the intestine [168]. Emerging evidence on migrants that increase intestinal permeability, however, means that higher-molecular-weight compounds must also be considered [165,169]. The magnitude of the issue is illustrated by FCCmigex, an open and continuously updated database created by Geueke et al. [170], which compiles data on several thousand compounds migrating from plastics, glass, paper, metals, composites, and other food-contact materials (FCM). Of special relevance to PP are the migration of polyolefin oligomers during storage and heating; the influence of contact time, temperature, and food composition (fats and acids) on stabiliser migration; and current approaches to detecting non-listed substances and assessing endocrine activity [165,166]. These issues shape the testing programme for biofilled composites and coatings.

Packaging–food interactions encompass permeation, migration, and sorption [166]. Migration denotes the transfer of chemical constituents—additives, plasticisers, residual monomers, oligomers, and contaminants—from the package into the food. Such transfer can compromise safety if the migrants are endocrine-active or carcinogenic, yet it can also be beneficial when antioxidants are released in a controlled way from active packaging. A distinction is drawn between overall migration, the total quantity of transferred substances without identification, and specific migration of individual identified compounds subject to their own limits. The process involves diffusion of a constituent through the material driven by a concentration gradient, desorption from the package surface, sorption at the interface, and subsequent transfer into the food, with the rate of each step depending on material composition, storage, and product characteristics [166].

Agricultural residues may carry pesticides, metals, extractives, and allergenic proteins, whereas aquatic wastes may contain salts, proteins, and organic contaminants; purification should therefore be confirmed analytically. Thermo-oxidation of PP and degradation of biomass and modifiers can generate NIAS that call for both targeted and screening analysis [165,166,171].

### Levels of Safety Evidence

A clear distinction should be made between a laboratory composite supported by mechanical data, an article with migration test results, and substantiated compliance for a defined formulation and use. Evidence at the first level does not establish the subsequent ones. Where a reviewed study reports no migration data, this signals a gap in the evidence base rather than demonstrated non-compliance. Likewise, the regulatory status of the base PP grade does not automatically extend to the fillers and coatings added to it.

Although eliminating plastics from daily life entirely is unrealistic, improper handling of packaging noticeably increases health risks [172]. Consumer awareness and labelling that encourages correct use are therefore important. Biodegradable, intelligent, and active packaging are regarded as means of reducing adverse impacts and improving food quality, but research on these technologies remains limited and their practical applicability is still debated [172].

## 6. Processing and Quality Control

The studies reviewed allow requirements to be formulated for the three stages of producing PP packaging materials with biofillers and coatings. Feedstock preparation includes drying the biofiller, controlling size distribution and ash content, chemically modifying surface hydroxyl groups with silanes, fatty acids, or maleated compatibilisers, and removing soluble extractives liable to migrate. Bulk composites are made by melt blending with MAPP, staged filler feeding, and antioxidants. Coatings require corona or plasma activation, waterborne primers, roll or slot-die application, drying under a mild temperature regime, and control of residual moisture. The test programme should cover mechanical and thermal properties, OTR and WVTR, transparency, heat sealability, microstructure (SEM), interfacial adhesion, and both overall and specific migration (OML, SML) under Regulation (EU) No 10/2011 using appropriate simulants; for active coatings, it is supplemented by in vitro bioactivity assessment and microbiological challenge testing.

The choice of biofiller is subordinate to the requirements of the finished package: mechanical strength, barrier, product preservation, seal integrity, and safety [171]. Biodegradability of an individual component does not override these requirements, and environmental benefits are assessed for a functionally equivalent article, taking recycling and food losses into account.

### 6.1. Regulatory Framework for Testing

The test programme should encompass the final architecture, including PP, coatings, adhesives, and ink layers, with raw-material specifications, production control, and migration testing linked to the intended use [150,163,164].

For PP subjected to radiation treatment for sterilisation or modification, the methodology must account for additional risks. Ionising radiation generates free radicals and reactive intermediates in the polymer, and the products of their conversion may accumulate with a delay. Even where overall compliance with Regulations No 1935/2004 and No 2023/2006 is achieved, this poses a risk of barrier deterioration and of exceeding the overall and specific migration limits of Regulation (EU) No 10/2011 [173]. For the irradiated PP/peanut-shell composites discussed above [73], migration testing should be conducted after irradiation and ageing rather than only on freshly prepared material.

Bringing together the requirements of Section 5 and Section 6 supports treating the testing of biofilled PP as a sequence tied to the article’s life cycle, not as a set of independent measurements. Mechanical, barrier, and migration properties must be determined on the same material in its final state—after processing, coating, irradiation where applicable [173], and accelerated ageing—since each stage can alter the interfacial zone, moisture content, and migrant profile. This sequence is particularly important for biofillers bearing extractives and a residual organic phase (Section 2.2.2 and Section 2.5), because these constituents are the most probable sources of non-listed migrants, whose detection requires dedicated analytical approaches [165,166].

### 6.2. Comparison with MOFs and Nanozymes

PP supplies the load-bearing matrix and is processed by well-established extrusion and moulding methods. Metal–organic frameworks (MOFs), by contrast, are viewed as porous carriers and adsorbents, and nanozymes as catalytic functional components [174,175]; neither is a direct substitute for the bulk PP matrix. For composites, cost is driven by waste preparation, additives, and drying, whereas for MOFs and nanozymes it additionally includes synthesis, purification, immobilisation, and preservation of activity. The data reviewed contain too few comparable industrial prices to allow a numerical ranking. A hybrid coating on PP could potentially combine these functions, but release of metals, ligands, and particles, effects on food, and recyclability would all need to be verified.

## 7. Environmental and Economic Aspects

Valorising waste does not in itself demonstrate a reduced environmental burden. Unfilled PP, PP with a conventional filler, and a waste-filled composite (including a coated variant) should be compared on the basis of an identical function, namely preserving a given quantity of food for a defined period (Table 7). The assessment accounts for article mass, feedstock collection and preparation, reagents, compounding, coating drying, rejects, food losses, and end-of-life management. System boundaries must include the existing route by which the waste is used or disposed of, and the burden of its preparation cannot automatically be set to zero [7,93,94,95].

The carbon footprint of waste-filled PP cannot be captured by a single reliable figure without an inventory of the specific formulation. For a preliminary comparison per 1 kg of a two-component material, Δg = w_f_(g_f_ − g_PP_) + Δg_proc_, where g_PP_ and g_f_ are the production emissions of PP and of the prepared filler, w_f_ is the filler mass fraction, and Δg_proc_ is the additional processing burden. A reduction is obtained only when the avoided PP burden exceeds the combined burden of waste preparation and additional processing. Comparing packages also requires accounting for density, thickness, coatings, food losses, and end of life; no measured carbon footprint has been established here for the formulations considered.

PP belongs to the technical cycle of materials, which involves reducing excess packaging, reusing where appropriate, and collection, sorting, and recycling. Adding waste does not automatically close this cycle; what matters are the recovery yield, property retention, and actual displacement of virgin PP. Changes in colour and odour, fibre shortening, accumulation of mineral residues, and coating incompatibility can result in downcycling, and any return to food contact requires its own safety justification [188,189].

## 8. Conclusions and Outlook

Material selection proceeds from defining the product and its storage conditions, through specifying mechanical, seal, and barrier requirements, to choosing a formulation and verifying the finished package. Cost and life-cycle performance are then compared with those of unfilled PP and of PP with a conventional filler at equal food preservation. Should no advantage be confirmed, the formulation or process route is revisited.

### 8.1. Concept of Functional Separation

Functional separation links mechanical support from the PP bulk, barrier action from a continuous coating, and product preservation from an active layer (Table 8). While it helps in selecting test metrics, it does not prove synergy, environmental superiority, or recyclability for an arbitrary multicomponent package. The barrier effect of filling should be measured rather than inferred solely from water absorption.

### 8.2. Quantitative Comparison of Biofillers

The specific modulus-gain index is defined as SMI_E_ = [100(E_c_ − E_0_)/E_0_]/c_f_, where E_c_ and E_0_ are the tensile moduli of the composite and of a comparable unfilled control, respectively, and c_f_ is the numerical filler content in wt.% (10 for 10 wt.%). It is expressed as percentage modulus gain per wt.% of filler. This is a descriptive normalisation, not the specific modulus E/ρ. A 136% gain at 40 wt.% gives SMI_E_ = 136/40 = 3.40, and an 85% gain at 10 wt.% gives 8.50 (Table 9). Flexural results are denoted SMI_F_ and treated separately.

Differences in SMI do not establish a universal ranking, because the studies differ in PP grade, particles, treatment, orientation, and test methods, and normalisation does not remove non-linear concentration effects. Strength retention, Rσ = 100σ_c_/σ_0_, and impact retention, RI = 100I_c_/I_0_, are calculated at the same loading and with the same method. Charpy and Izod values, whether notched or unnotched, are not combined, and the indices are not calculated where paired data are lacking.

For a void-free two-phase system, mass and volume fractions are related by φ_f_ = (w_f_/ρ_f_)/[(w_f_/ρ_f_) + (1 − w_f_)/ρ_PP_]. With ρ_PP_ = 0.90 and ρ_f_ = 2.50–2.71 g/cm^3^, a content of 20 wt.% corresponds to 7.7–8.3 vol.% and 40 wt.% to 18.1–19.4 vol.%. This conversion describes the formulation and does not by itself identify an environmentally optimal loading.

### 8.3. Assessment of the Evidence Base and Sources of Discrepancy

Data on the mechanical and thermal properties of biofilled PP are the most extensive, whereas combined measurements of OTR, WVTR, migration, and recyclability are considerably rarer. Discrepancies arise from feedstock preparation, matrix grade, interfacial modification, and test conditions. Meaningful comparison requires control samples within the same study and explicit identification of missing data.

To remedy these shortcomings, we propose a minimum dataset to be reported in studies of biofilled PP:

1. Waste origin, isolation and pretreatment method, moisture content, ash content, and particle-size distribution, plus organic-phase content and polymorph composition for bio-CaCO_3_;
2. Compatibiliser type and dosage, expressed as a fraction of matrix mass;
3. Filler content as both mass and volume fractions;
4. Impact test method, with the standard specified;
5. OTR and WVTR, with the measurement temperature and relative humidity;
6. Migration from the finished article in all simulants and under all conditions relevant to the intended use, including assessment of specific contaminants and NIAS.

### 8.4. Economic Feasibility and Scale-Up

Economic feasibility should be judged by the cost of a conforming packaging article. The PP saved must offset the collection, cleaning, drying, and grinding of the waste, the compatibiliser, additional operations, quality control, and rejects, and any increase in wall thickness must also be taken into account. For bio-CaCO_3_, a control with commercial CaCO_3_ is essential. Feedstock variability and insufficient preparation infrastructure constrain scale-up [7]. Throughput, energy consumption, and yield of conforming product should be measured on a pilot line. A coating is justified when its functional effect is confirmed and weighed against the added cost and the impact on recycling. The available data do not define a general profitability threshold for such systems.

### 8.5. Outlook

The way forward lies in identifying the conditions under which a particular formulation confers an advantage on the finished article. Filler content and the need for a coating are dictated by product requirements, and no universal optimum has been established. Priorities include comparisons on a single PP grade, barrier performance after humid storage and flexing, migration, food trials, and pilot-scale assessment of cost, energy, and recyclability:

- Rice straw cellulose should be compared as a bulk filler and as a coating component for the same packaging function;
- The longer oxidation induction period of PP containing olive pits [65] reflects polymer stabilisation, and preservation of the food product must be confirmed separately;
- Bio-CaCO_3_ particles with a controlled polymorph composition (Section 2.2.2) and porous natural carriers (Section 3.4.3) could be combined into hybrid fillers acting as reservoirs of active agent within the PP bulk, provided that the migration issues (Section 2.2.3) are resolved;
- The moisture resistance of nanocellulose and chitosan coatings should be evaluated in laminates at realistic storage humidity rather than in free-standing films at low humidity.

In summary, whether it is worthwhile to modify PP with components from agricultural and aquatic waste is decided by the benefits delivered by a specific packaging article, not by the mere use of secondary raw materials. Savings in virgin polymer and improvements in properties must be weighed against the cost of filler preparation, package mass, safety, and recyclability. The studies reviewed confirm the potential of individual formulations but do not demonstrate their general economic or environmental superiority. The priority for future research is to define the conditions under which such systems offer measurable advantages over unfilled PP and PP with conventional fillers at equal preservation of the food product.

## Figures and Tables

**Table 1:** Chemical composition of rice husk [16].

Component	Content, wt.%
Cellulose	25–35
Hemicellulose	18–21
Lignin	26–31
SiO_2_	15–17
Soluble matter	2–5
Moisture	5–10

**Table 2:** Waste-derived components in PP: (a) formulation and processing; (b) properties. NV—information not verified against available data; missing values were not interpolated.

**(a) Formulation and Processing**				
**Code and Source**	**Filler; Size**	**Loading, wt.%**	**Modification and PP Grade**	**Processing**
RS0 [21]	None	0	PP; commercial grade NV	Melt blending; specimen data NV
RS10 [21]	Rice straw cellulose; size NV	10	Alkaline isolation and H_2_O_2_ washing; grade NV	Melt blending
RS20 [21]	Rice straw cellulose; size NV	20	Same study; compatibiliser not verified	Melt blending
RS50 [21]	Rice straw cellulose; size NV	50	Same study; grade NV	Melt blending
ES40 [25]	Eggshell; <38 μm	40	Unmodified; grade NV	NV in extracted data
SS10 [25]	Mollusc shell; <200 nm	10	4% NaOH treatment; grade NV	Twin-screw compounding and injection moulding
SN30 [53]	Snail shell; <10 μm	30	Modifier and grade NV	NV in extracted data
OP [59,65]	Olive pits; size source-dependent	5–20 [65]	Maleic anhydride treatment reported; grade NV	NV in extracted data
RH [17,18]	Rice husk; size NV	Source-dependent; no general optimum	MAPP studied; grade NV	Extrusion and moulding; formulation-dependent
**(b) Mechanical and Barrier Characteristics**				
**Code**	**Strength, MPa**	**Young’s Modulus, MPa**	**Elongation, %**	**OTR/WVTR**
RS0	34.3	638.5	57.5	NV/NV
RS10	30.1	686.4	15.2	NV/NV
RS20	28.1	731.3	10.9	NV/NV
RS50	21.4	824.2	4.5	NV/NV
ES40	NV	Absolute value NV; +136% vs. control	NV	NV/NV
SS10	NV at 10 wt.%	Absolute value NV; +85% vs. control	NV	NV/NV
SN30	Absolute and exact paired values NV	Absolute value NV; approx. +100%	NV	NV/NV
OP	NV	NV; increase reported	NV	NV/NV
RH	NV	NV	NV	NV/NV

**Table 3:** Layer functions in a multilayer coating on PP.

Layer	Function
PP substrate	Mechanical support
Activated surface	Adhesion enhancement
Primer	Interfacial compatibility
Barrier layer	Reduction of gas and water-vapor permeability
Protective layer	Mechanical and chemical protection

**Table 4:** Comparison of waterborne primer systems for PP films.

Primer Type	Advantages	Disadvantages	Application Area
Polyurethane (WPU)	High adhesion, flexibility	High cost	Printing, lamination
Acrylic	Transparency, UV resistance	Lower adhesion to PP	Print coatings
PU–acrylic hybrid	Balanced properties	Complex synthesis	Barrier coatings
Silane-modified	High adhesion	Moisture sensitivity	Functional coatings

**Table 5:** Barrier characteristics of specific systems and measurement conditions. NV—not verified against available data; this does not imply a zero value or that the information is necessarily absent from the original source.

Study and Substrate	Layer and Thickness	Oxygen Metric	Conditions and Method	WVTR
Lu et al. [109]; BOPP/LDPE	Uncoated control; thickness NV	OTR 67.03 cm^2^·m^−2^·day^−1^	23 °C; 50% RH; Mocon OXTRAN 2/21; standard edition NV	NV
Lu et al. [109]; BOPP/LDPE	NC coating; dry thickness NV	OTR 24.02 cm^2^·m^−2^·day^−1^	Same-study conditions as for control	NV
Sharbafian et al. [115]; CPP70	Uncoated control; nominal substrate thickness 70 μm	Permeance 1516 cm^2^·m^−2^·day^−1^·bar^−1^	23 °C; 50% RH; ASTM D3985 as reported by the authors; Labthink C230M	NV
Sharbafian et al. [115]; CPP70	Semi-industrial CBS004 coating; dry thickness NV	Permeance 36 cm^2^·m^−2^·day^−1^·bar^−1^	Same-study conditions as for control	NV

**Table 6:** Potential risks of PP fillers and coatings and the checks they require. The existence of a risk does not mean that it is present in every batch.

Source or Operation	Possible Risk	Verification on the Finished Article
RH, RS, fruit and nut residues	Pesticides, metals, extractives, residual allergenic proteins.	Batch traceability; targeted contaminant analysis; protein analysis where needed; application-specific migration.
Eggshells and mollusc shells	Residual proteins, metals, salts, biological contamination.	Confirmed purification and deproteinisation; elemental and organic screening; acidic-contact testing where needed.
Isolation of chitosan and nanocellulose	Residual acids and alkalis, solvents, bleaching agents, low-molecular-weight products.	List of process reagents, purity specifications, and migrant analysis.
Extrusion, irradiation, ageing, and recycling	PP oxidation products; degradation products of biomass, stabilisers, and modifiers; NIAS.	Screening after the final thermal or radiation history and ageing; identification of likely migrants and exposure assessment.
Active coatings and hybrid particles	Intended release of the agent; unintended transfer of linker, metal, and particles; sensory effects.	Separation of release kinetics from migration safety; evaluation of the whole article under the intended food contact [150,151,165,166].

**Table 7:** Major initiatives aimed at reducing the environmental impact of plastic packaging.

Organisation/Region	Documents and Stated Initiatives	Source
UNEP	Resolution UNEA-5/14 (2022) on developing an international legally binding instrument on plastic pollution.	[176]
European Union	Directive (EU) 2019/904; chemicals and circular-economy strategies (2020); Regulations (EU) 2025/40 (PPWR), 2024/3190 (bisphenol A), 2024/1781 (ESPR).	[177,178,179,180,181,182]
United States	National Recycling Strategy (2021; 50% target by 2030); draft strategy to prevent plastic pollution (2023).	[183,184]
China	Plastic pollution control plan for 2021–2025: restriction of single-use items, recycling, and alternatives.	[185]
Japan	Act on Promotion of Resource Circulation for Plastics, in force since April 2022.	[186]
Australia, New Zealand, Pacific Islands	ANZPAC Plastics Pact (2021): commitments to eliminate problematic packaging and a target of 100% reusable, recyclable, or compostable packaging by 2025; attainment of this target is not assessed here.	[187]

**Table 8:** Functional levels of PP packaging with waste-derived components: assessment of the state of research.

Functional Level	Candidate Components	Evidence and Limitations
PP-dominated bulk	RH, RS, pits, shells, bio-CaCO_3_	Stiffness may increase, whereas strength, ductility, and impact resistance may decrease. Substitution with waste is not the outcome of a carbon-footprint calculation.
Barrier layer	Nanocellulose, nanochitin, PVOH, or hybrids	OTR reduction confirmed within individual studies [97,109,115]; transferability limited by humidity, defects, and dry coat weight.
Active layer	Chitosan, nisin, polyphenols, and carriers	Activity depends on method, dose, and release. Polymer stabilisation [65] does not prove food preservation.
Whole article	Combined formulation and architecture	Synergy, migration compliance, shelf life, and recycling require joint verification; no general claim of compliance is made.

**Table 9:** Descriptive normalisation of the efficiency of carbonate fillers in PP based on [25,53]. NV—not verified for the same formulation and filler content. Estimates taken from review [25] require verification against the primary studies.

Filler and Source	Size; Loading	Modulus Gain	Index	Paired Strength and Impact Data
Eggshell [25]; secondary estimate	<38 μm; 40 wt.%	Tensile +136%	SMI_E_ 3.40	Rσ NV; RI NV
Eggshell [25]; secondary estimate	14–157 μm; 40 wt.%	Tensile +220%	SMI_E_ 5.50	Rσ NV; RI NV
Alkali-treated mollusc shell [25]; secondary estimate	<200 nm; 10 wt.%	Tensile +85%	SMI_E_ 8.50	Rσ at 10 wt.% NV; RI NV
Mollusc shell [25]; separate flexural measurement	2–4 μm; 30 wt.%	Flexural +62%	SMI_F_ 2.07	Rσ and RI for the same formulation NV
Snail shell [53]; approximate relative data	<10 μm; 30 wt.%	Tensile +100%	SMI_E_ 3.33	Strength retention across series 91–100%; exact paired value NV; RI NV

## Data Availability

No new data were created or analyzed in this study. Data sharing is not applicable to this article.

## Abbreviations

The following abbreviations are used in this manuscript:

BOPP	biaxially oriented polypropylene
CFU	colony-forming units
CNC	cellulose nanocrystals
CNF	cellulose nanofibers
CPP	cast polypropylene
DMA	dynamic mechanical analysis
DTG	differential thermogravimetry
ECC	engineered cementitious composites
EFSA	European Food Safety Authority
ENR	epoxidized natural rubber
EO	essential oil
EVOH	ethylene–vinyl alcohol copolymer
FCM	food contact material
FTIR	Fourier-transform infrared spectroscopy
HDPE	high-density polyethylene
LCA	life cycle assessment
LDPE	low-density polyethylene
MAPP	maleic anhydride-grafted polypropylene (PP-g-MA)
MFC	microfibrillated cellulose
MMT	montmorillonite
NC	nanocellulose
OML	overall migration limit
OPP	oriented polypropylene
OTR	oxygen transmission rate
PAE	phthalic acid esters
PE	polyethylene
PEAA	poly(ethylene-co-acrylic acid)
PET	poly(ethylene terephthalate)
PHA	polyhydroxyalkanoate
PLA	poly(lactic acid)
PP	polypropylene
PPC	poly(propylene carbonate)
PS	polystyrene
PVC	poly(vinyl chloride)
PVOH	poly(vinyl alcohol)
RH	rice husk
RHA	rice husk ash
RHF	rice husk fiber
RS	rice straw
SCG	spent coffee grounds
SEBS	styrene–ethylene/butylene–styrene
SEM	scanning electron microscopy
SML	specific migration limit
SS	seashell (mollusk shell)
UV	ultraviolet
WPU	waterborne polyurethane
WVP	water vapor permeability
WVTR	water vapor transmission rate
